# Xanthine oxidoreductase in cancer: more than a differentiation marker

**DOI:** 10.1002/cam4.601

**Published:** 2015-12-21

**Authors:** Maria Giulia Battelli, Letizia Polito, Massimo Bortolotti, Andrea Bolognesi

**Affiliations:** ^1^ Department of Experimental, Diagnostic and Specialty Medicine – DIMESAlma Mater Studiorum – University of Bologna, General Pathology UnitVia S. Giacomo 1440126BolognaItaly

**Keywords:** Differentiation, oncogenesis, reactive oxygen and nitrogen species, uric acid, xanthine oxidoreductase

## Abstract

Human xanthine oxidoreductase (XOR) catalyzes the last two steps of purine catabolism and is present in two interconvertible forms, which may utilize O_2_ or NAD ^+^ as electron acceptors. In addition to uric acid, XOR products may comprise reactive oxygen and nitrogen species that have many biologic effects, including inflammation, endothelial dysfunction, and cytotoxicity, as well as mutagenesis and induction of proliferation. XOR is strictly modulated at the transcriptional and post‐translational levels, and its expression and activity are highly variable in cancer. Xanthine oxidoreductase (XOR) expression has been negatively associated with a high malignity grade and a worse prognosis in neoplasms of the breast, liver, gastrointestinal tract, and kidney, which normally express a high level of XOR protein. However, the level of XOR expression may be associated with a worse outcome in cancer of low XOR‐expressing cells, in relation to the inflammatory response elicited through the tissue damage induced by tumor growth. Xanthine oxidoreductase (XOR) has been implicated in the process of oncogenesis either directly because it is able to catalyze the metabolic activation of carcinogenic substances or indirectly through the action of XOR‐derived reactive oxygen and nitrogen species. The role of uric acid is characterized by both oxidant and antioxidant action; thus, it is still debatable whether control of uricemia may be helpful to improve the outcomes of tumor illness.

## Introduction**:** Biology of Xanthine Oxidoreductase

**Figure 1:**
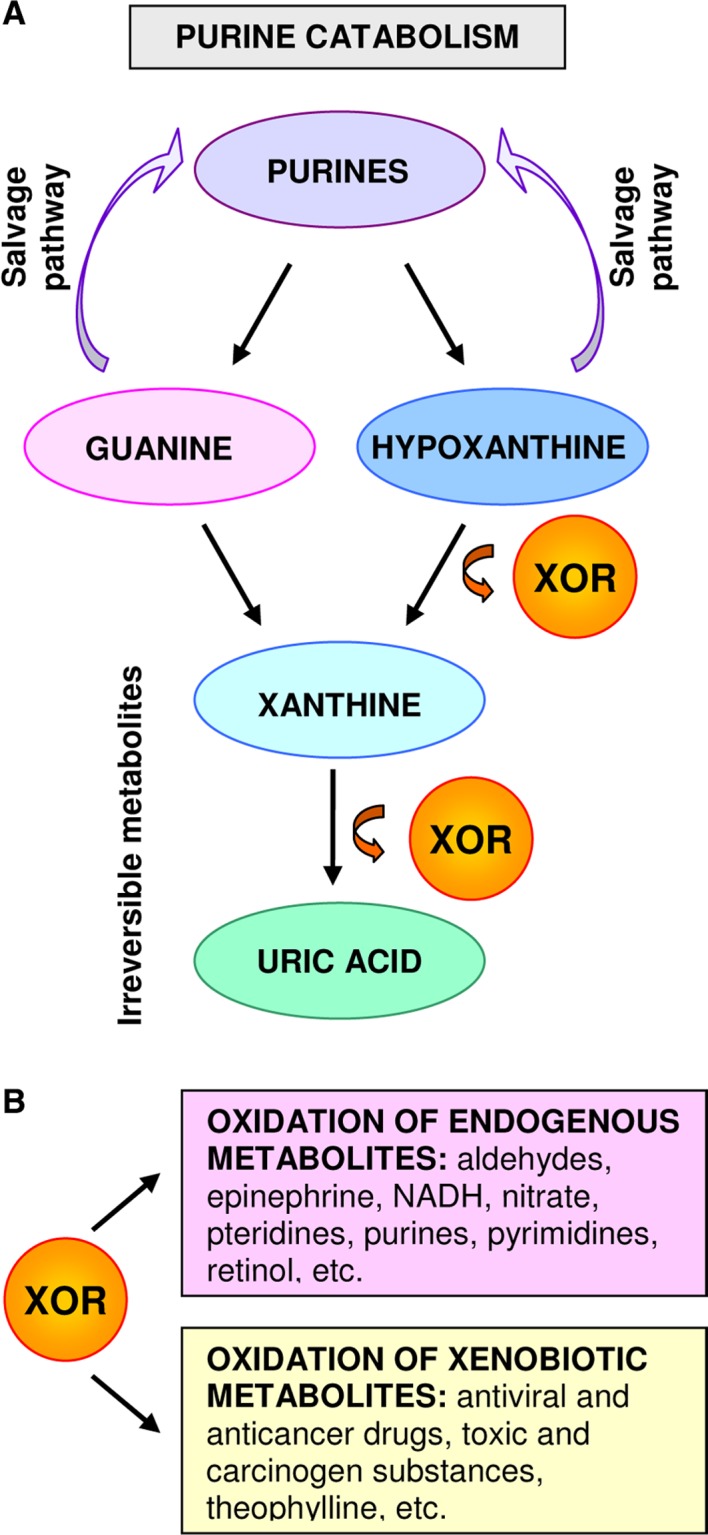
Substrates of Xanthine oxidoreductase (XOR) activity. (A) XOR catalyzes the last two steps of purine catabolism and generates xanthine and uric acid, therefore precluding the salvage pathway of purine nucleotides. (B) XOR can oxidize different endogenous metabolites, including NADH and nitrate, thus producing reactive species of both oxygen and nitrogen. XOR can also oxidize various xenobiotics, hence activating both carcinogens and anticancer drugs.

Xanthine oxidoreductase catalyzes the oxidation of hypoxanthine to xanthine and of xanthine to uric acid, that is, the last two steps of purine catabolism in the highest uricotelic primates. In addition, XOR can oxidize different endogenous metabolites as well as various xenobiotics, including toxic substances and anticancer drugs (reviewed in 1). By generating irreversible products, XOR has a rate‐limiting role in purine metabolism because its action precludes the salvage pathway of purine nucleotides (Fig. 1).

Xanthine oxidoreductase is a homodimeric metalloflavoprotein with a molecular mass of approximately 300 kDa. Each subunit includes a molybdenum‐containing molybdopterin cofactor, two nonidentical iron–sulfur redox centers, and one flavin adenine dinucleotide (FAD) cofactor (reviewed in 2). Low levels of XOR activity are almost ubiquitous in mammalian tissue, with the highest levels present in the liver, intestine, kidney, lactating mammary gland, and vascular endothelial cells (reviewed in 3).

Xanthine oxidoreductase is strictly regulated at the transcriptional and post‐translational levels. The expression of XOR protein is increased by various hormones, growth factors, inflammatory cytokines, irritative stimuli, and low oxygen tension. The post‐translational modulation of XOR consists of quantitative and qualitative changes in its activity. Interconvertible demolybdo‐ and/or desulfo‐forms of XOR protein are known to be inactive in xanthine catalysis, but they may still oxidize NADH. XOR activity may also be controlled through the switch between XOR dehydrogenase and oxidase forms. Such conversion may occur in different pathologic circumstances, including tissue damage by physical agents, poisoning with endogenous or toxic substances, and various hypoxic/ischemic conditions (reviewed in 4).

**Figure 2:**
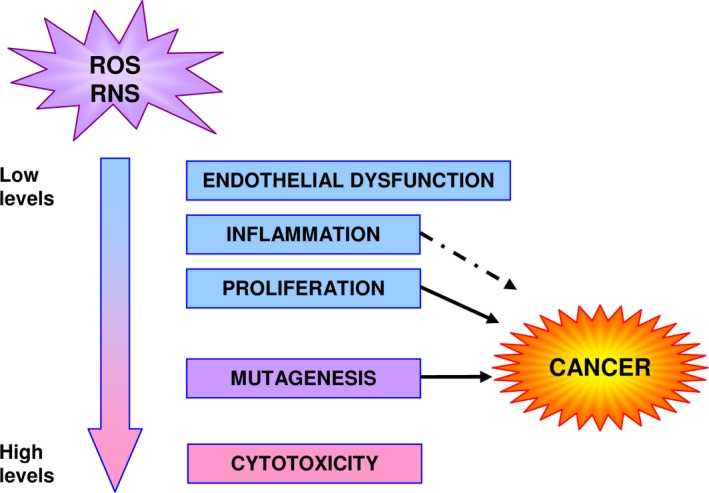
Biologic effects of reactive oxygen species (ROS) and reactive nitrogen species (RNS) and their implication in cancer pathogenesis. Low levels of ROS and RNS may stimulate the inflammatory response, endothelial dysfunction, and cell proliferation, whereas higher levels may induce mutagenesis or have cytotoxic effects. The arrows indicate effects directly (continuous line) or indirectly (dashed line) related to oncogenesis.

Xanthine oxidoreductase is constitutively expressed as xanthine dehydrogenase (XDH, EC 1.1.1.204), which transfers the electrons to NAD^+^. The final product of XOR activity, uric acid, has an antioxidant action in the extracellular environment, providing protection in the blood circulation against oxidative stress. However, in mammals, XOR may be converted to xanthine oxidase (XO, EC 1.1.3.22) through the oxidation of sulfhydryl residues or limited proteolysis. XO utilizes molecular oxygen as an electron acceptor and produces the reactive oxygen species (ROS) superoxide anion and hydrogen peroxide. XOR can generate ROS also by oxidizing NADH, and can contribute to the formation of reactive nitrogen species (RNS) by converting nitrites into nitric oxide. Both ROS and RNS may have many biologic effects, including endothelial dysfunction, inflammation, induction of proliferation, mutagenesis, and cytotoxicity (reviewed in 4, 5) (Fig. 2).

## Level of Xanthine Oxidoreductase in Cancer Tissue

### Activity in animal tumors

The first detection of XOR in neoplastic tissues occurred 80 years ago in the extracts of some rodent tumors 6.

Thereafter, a very low XOR activity was found in rat liver during carcinogenesis induced by p‐dimethylaminoazobenzene and a low‐protein diet, while a parallel increase in serum enzyme was observed, suggesting a “leakage” of the liver enzyme into the blood stream 7. After feeding rats for a short period with carcinogenic azo‐dyes, a decrease in XOR activity level was somewhat present in precancerous liver and was remarkable in the resulting hepatoma 8. Diethylnitrosamine by intraperitoneal injection induced hepatocellular carcinoma in rats. During the oncogenesis process, a significant XOR decrease in hepatic tissue was observed compared with that in liver of untreated rats 9. Additionally, XOR activity was lower in rapidly growing transplantable hepatocellular carcinoma HC‐252 than in resting normal rat liver tissue 10.

The catabolism of purines in rat Novikoff hepatoma was deeply impaired, and no activity of XOR could be detected 11. Accordingly, in chemically induced transplantable hepatomas in rat, the activities of the anabolic purinic enzymes increased, whereas the activities of catabolic enzymes, including XOR, decreased 12. This alteration may be functional to cancer cell growth because it allows the recycling of purines through the salvage pathway for nucleic acid biosynthesis 13 (reviewed in 14).

Xanthine oxidoreductase activity progressively decreases also during carcinogenesis in mouse breast tissue 15. In particular, the XOR level in breast tissue of a low‐tumor strain was twice than in that of a high‐tumor strain and was further halved in spontaneous mammary tumors 16.

However, the variation in XOR level is not strictly dependent on cell proliferation, since XOR activity increased markedly in mouse breast during pregnancy and lactation 17, 18, as well as in rat liver during postnatal growth 13, 19. Also, no changes were reported in XOR activity of regenerating rat liver compared with the liver of sham‐operated controls 13, 19. Moreover, as compared with normal rat liver, a 2‐ to 10‐fold decrease in XOR activity was reported in various hepatoma cell lines, irrespectively of the growth rate or degree of histologic differentiation 13, 19. At a contrast with the precedent observation, the XOR activity decrement in the rapidly growing Morris hepatoma 3924A was higher than in the slowly growing chemically induced transplantable hepatoma 20, being 4 and 34% of those of normal rat liver, respectively 20.

As compared with the levels in normal intestinal tissue, significantly lower levels of XOR activity have been reported in mouse colon adenocarcinoma 21, as well as in chemically induced tumors of the rat large bowel 22. XOR activity in the serum of treated rats was considerably higher than that of the control animals 22. A metabolic imbalance was reported in mouse colon adenocarcinoma favoring purine synthesis over degradation, in a manner similar to that observed in rat hepatomas 21.

Similar biochemical alterations were described in rat kidney tumors 23, as well as in a rapidly growing rat rhabdomyosarcoma, with the level of XOR activity at 54% compared with that in muscle tissue, independently of the rate of cell replication because XOR activity was doubled in a differentiating muscle compared with adult muscle 24.

Contrasting results were obtained with murine skin during carcinogenesis, and in papillomas and squamous cell carcinomas induced by 12‐O‐tetradecanoylphorbol‐13‐acetate. The activities of several antioxidant enzymes were reduced, whereas XOR activity was significantly increased, as measured in age‐matched, nontreated mice or in skin adjacent to the tumors 25. These findings are somewhat in agreement with the results obtained with the transplantable Guerin's epithelioma in rats. The level of XOR activity and, in particular its oxidase form, increased during the period of carcinoma active growth together with the formation of ROS. XOR activity declined at the terminal stages of oncogenesis, when the enzyme protein was degraded, suggesting a role for ROS in the mechanism of tumor pathogenesis 26.

In most of the cases, XOR expression and activity decreased in tumor tissues compared with the corresponding normal ones. The experimental reports are generally focused on the liver, breast, colon, and kidney cancers, that is, tumors derived from the tissues with the highest XOR activity. Thus, it is not surprising that a lack of differentiation in malignant neoplasms may correspond to a low level of a highly expressed protein such as XOR. However, in tumors derived from low XOR‐expressing tissues, such as skin squamous cell carcinomas, other considerations should be made. For instance, an inflammatory response with an increase in the XOR activity level could be expected.

### Activity in human tumors

In two lines of human colon carcinoma xenografts, which were carried in nude mice, purine metabolism appeared to depend on the different growth rates. The rapidly growing carcinoma cells showed a higher level of purine biosynthetic enzymes and a lower level of purine catabolic enzymes compared with the slower growing cell line. Particularly, the rate‐limiting XOR activity in the rapidly growing and poorly differentiated colon cancer was decreased to 17% compared with the slower growing, well‐differentiated tumor 27. Additionally, XOR activity decreased, whereas the level of enzymes involved in the purine salvage pathway increased, in cancerous human colon tissues compared with noncancerous ones, conferring selective growth advantages to neoplastic tissues 28.

Xanthine oxidoreductase activity was significantly decreased in primary renal cell carcinomas compared with control kidney cells 23. Similar alterations of the purine metabolism were observed by comparing cancerous and noncancerous human tissues from patients with bladder cancer 29 or renal cell carcinoma 30, suggesting an increase in salvage pathway activity in tumor cells.

The activity of the dehydrogenase form of XOR was decreased, although its oxidase form was increased, in human liver cancers compared with noncancerous human liver tissues. The free radical defense enzymes were found to be depressed, and the DNA turnover was accelerated in the same samples 31. Accordingly, with the above findings, XOR activity was significantly lower in neoplastic than in control tissue, as well as in a nontumoral area of the same liver, in patients with hepatocarcinoma 32.

Low levels of XOR and cellular retinol binding protein have been reported in malignant human mammary epithelial cells compared with normal ones. The consequent retinoic acid deficiency could be responsible for the lack of differentiation and may be implicated in mammary carcinogenesis 33.

Dissimilar results were obtained in patients with tumors of different histologic origin. Significantly higher XOR levels were observed in meningioma and astrocytoma from the human brain compared with normal brain tissue 34. Increased XOR activity was also reported in human laryngeal well‐differentiated squamous cell carcinomas compared with the corresponding tumor‐free adjacent tissues or normal laryngeal tissues 35.

Increased activities of the enzymes participating in purine metabolism, including XOR activity, have also been described in cancerous human colorectal tissues, whereas the activities of free radical‐metabolizing enzymes decreased compared with those of noncancerous adjacent tissues. The increment in XOR activity was interpreted as a reaction to accelerated DNA turnover, while the reduced antioxidant defense could be, at least in part, responsible for the carcinogenic processes 36.

In cancerous human prostate tissue, compared with noncancerous tissues, the activities of DNA turnover enzymes decreased, and the activities of free radical‐metabolizing enzymes increased, although the activities of XOR and superoxide dismutase were unchanged. The overall results suggested that a low rate of purine catabolism, as well as an increased free radical stress occur in tumoral prostate tissues 37.

Finally, as sometimes reported in tumor‐bearing animals, an elevated level of XOR activity was also observed in the plasma of patients with different types of cancer 38.

### Expression in human tumors

Normal human breast has been shown to express XOR protein in the cytoplasm of the epithelium lining terminal ducts. XOR expression was markedly enhanced in the alveolar epithelium of lactating mammary lobules. No detectable XOR was observed either in situ or in invasive carcinomas of the breast 39. In agreement with these results, XOR expression was strong in normal breast epithelium and was expressed at different levels in 1262 breast cancers, independently from the hormone receptor status of the tumor. A low level of expression was found in large‐sized cancers with poor histologic grade of differentiation and a high number of positive axillary lymph nodes. The loss of XOR expression was associated with an unfavorable prognosis. Indeed, the risk of distant recurrence was doubled in patients with no XOR expression compared with those with a moderately decreased or normal expression. Thus, the absence of XOR expression was an independent prognostic factor, associated with unfavorable outcomes. However, dedifferentiation did not invariably lead to loss of XOR 40. Moreover, the XOR level was found to be higher in breast cancer cell lines with no or low migratory capability than in highly invasive mammary tumor cells, suggesting a potential role for XOR in suppressing breast cancer pathogenesis 41.

Xanthine oxidoreductase expression in 337 human gastric cancers varied from normal to undetectable levels compared with the corresponding normal tissue. Downregulation of XOR expression was associated with the parameters of a more aggressive gastric cancer in terms of cellular anaplasia, mitotic index, invasiveness, tumor size, and lymph node metastasis. Low XOR expression resulted in a marker of unfavorable outcome and poor specific survival in patients with gastric cancer 42.

Similar results were obtained by comparing the level of XOR expression in human colorectal cancer and in normal intestinal tissue of 478 patients. A variable decrease was observed in most of the tumor samples; however, poor XOR expression was associated with a low grade of histologic differentiation and a more advanced cancer stage. Moreover, no XOR expression was detected in vitro in the undifferentiated Caco‐2 cell line, derived from human colon adenocarcinoma, but XOR expression appeared and increased when these cells spontaneously differentiated during cell culture. Thus, the level of XOR expression may also be a prognostic factor for the clinical outcomes of colorectal carcinomas, suggesting a neoplasia suppressor role for XOR in this tumor, as well as in breast and gastric cancers 43.

Decreased XOR expression in tumor tissue compared with the corresponding normal tissue was associated with a worse prognosis in 474 patients with serous ovarian carcinoma; XOR expression was markedly lower in poorly differentiated tumors. Moreover, the outcome and specific survival were negative, even in cancers with an otherwise more favorable prognostic profile, when XOR was absent 44.

The expression of XOR protein was absent in normal human lung tissue, while it was observed at various levels in 65% of different types of non‐small‐cell lung cancers. Patients with a high percentage of cells expressing XOR protein had a longer median survival than patients with a lower expression frequency. Additionally, a low intensity of XOR expression was associated with a worse prognosis for patients who received adjuvant chemotherapy, suggesting a role for XOR‐produced ROS in contributing to the tumoricidal effect of chemotherapy 45. While the lack of XOR expression was confirmed in human lung tissue, the acquisition of such expression was associated with a shortened survival in patients with resected lung adenocarcinoma. Thus, the level of XOR mRNA in tumor tissue was correlated with the worsening of patient outcomes, suggesting it to be an independent predictor of a poor prognosis 46.

**Figure 3:**
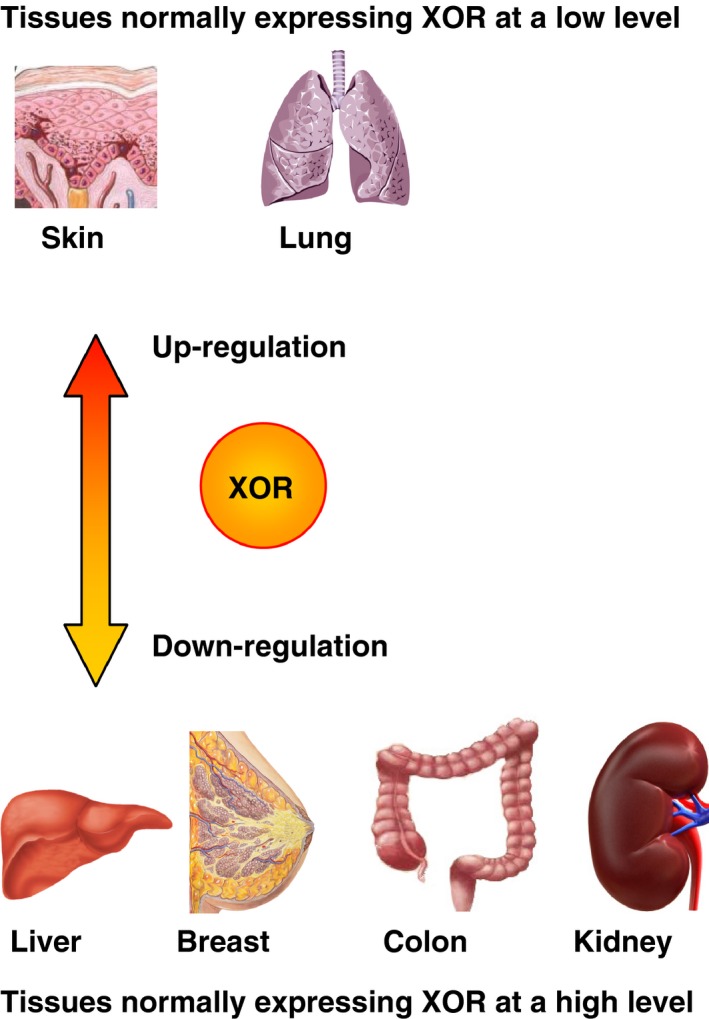
Expression and activity of Xanthine oxidoreductase (XOR) in cancer. The level of XOR expression and activity in cancer tissue was down‐ or upregulated in the malignancies of tissues, which express XOR protein at high (liver, breast, colon, and kidney) or low (skin and lung) levels, respectively.

A higher variability related to the XOR level was observed in the clinical results compared with the experimental ones. This variability cannot be unexpected for obvious reasons because the laboratory animals have more homogeneous genetic material. However, low XOR expression was associated with a high malignity grade and a worse prognosis in patients with breast, liver, gastrointestinal, and kidney cancers, which are derived from tissues expressing a high level of XOR protein. Similar to what was observed in animal tumors, an increased XOR level in low‐expressing tissues, as well as the acquired XOR expression in the usually not expressing lung tissue, may have a divergent meaning, possibly indicating an inflammatory reaction, in particular being associated with the aggravation of patient outcome (Fig. 3).

## Role of Xanthine Oxidoreductase in Differentiation and Oncogenesis

### Development and differentiation

**Figure 4:**
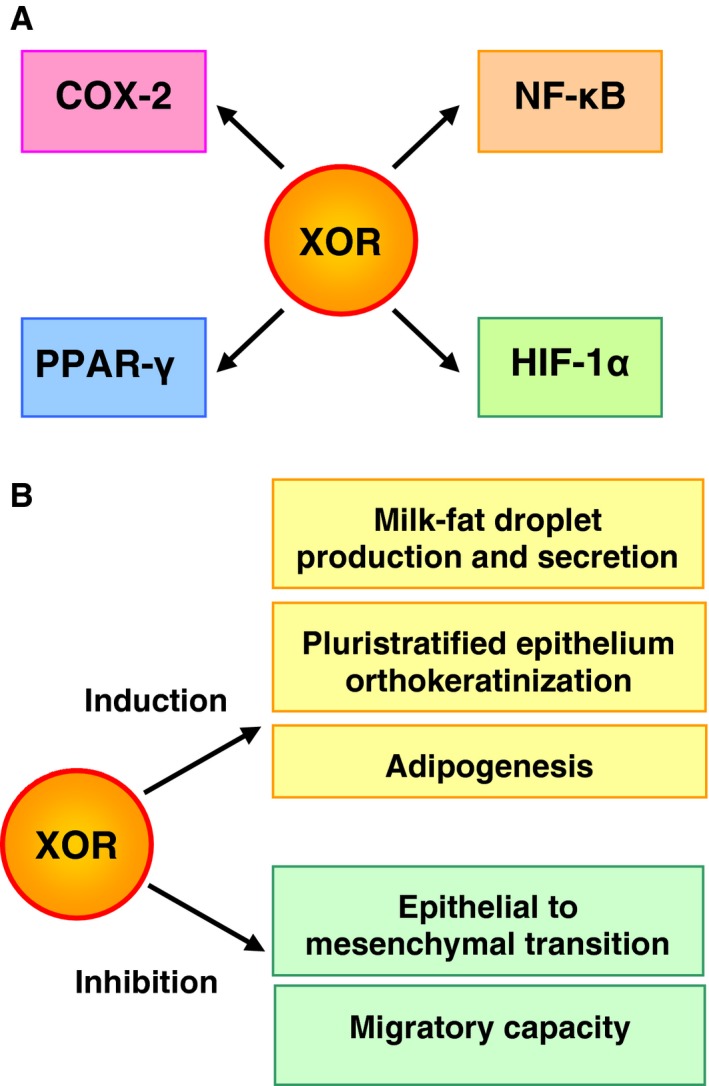
Molecular and cellular actions of Xanthine oxidoreductase (XOR). (A) Signal transduction molecules upregulated by XOR. COX‐2 is an inducible enzyme producing pro‐inflammatory metabolites of arachidonic acid, which are involved in oncogenesis and progression to malignancy by promoting cell proliferation and migration. COX‐2 expression requires the phosphorylation of NF‐*κ*B and could be inhibited by XOR 41, but stimulated by uric acid 47. NF‐kB is a transcription factor that may be activated by XOR‐produced reactive oxygen species (ROS) 48, 49. NF‐kB activity is a hallmark of chronic inflammation and cancer, because its signaling pathway maintains high expression of a set of antiapoptotic genes. HIF‐1*α* is a transcription factor implicated in cancer development and progression by promoting the expression of angiogenic target genes, including vascular endothelial growth factor. In normoxic conditions, activated NF‐kB contributes to the expression and activity of HIF‐1*α* together with ROS, including XOR‐derived ROS 50. Peroxisome proliferator‐activated receptor gamma (PPAR)‐*γ* is a ligand‐activated intracellular transcription factor, included in the nuclear hormone receptor superfamily that has an antitumorigenic and antiproliferative action by favoring cell differentiation and inhibiting angiogenesis. XOR stimulated the activation of PPAR‐*γ* through the ROS produced by its NADH‐oxidizing activity 51. (B) Cell differentiation aspects affected by XOR. Milk fat droplet production and secretion requires the formation of a complex between XOR, adipophilin and butyrophilin 53. Thus, in lactating mammary epithelium of mice, XOR expression is essential to maintain lactation 52, possibly because XOR regulates the binding of fat droplets to the apical cell membrane and their secretion 18. Pluristratified epithelium orthokeratinization was associated to XOR expression and conversion of xanthine dehydrogenase (XDH) to xanthine oxidase (XO) in ovariectomized mice after hormonal induction of vaginal epithelium hyperplasia, suggesting that XOR activity is required for the orthokeratinization pathway of differentiation 55. Adipogenesis is controlled by a cascade of factors that includes XOR as a regulator of the adipogenic process. Indeed, in vivo the level of XOR expression correlates with fat accumulation in mice and XOR activity was linked to the adipogenic transcription factor PPAR‐*γ* 51. Epithelial to mesenchymal transition was observed in primary renal epithelial cells from *XOR*−/− mice 54. Not only the cell morphology changed from a cuboidal to a myofibroblastic shape, but also immunohistochemical analysis showed a positive staining for markers of mesenchymal cell type, suggesting that XOR expression is needed for epithelial differentiation in mouse kidney. Migratory capacity in vitro was inversely correlated with XOR expression in epithelial cell lines derived from breast cancer 41.

Despite some controversial results, the alteration of XOR expression and activity, both in experimental and clinical reports, suggested a role for XOR in the differentiation process. Indeed, XOR has been implicated in cell differentiation because of its ability to regulate various molecules involved in the transduction of inter‐ and intracellular signals: (1) pro‐inflammatory cyclooxygenase‐2 (COX‐2) 41, 47, (2) transcription factor NF‐*κ*B (nuclear factor kappa‐light‐chain‐enhancer of activated B cells) 48, 49, (3) hypoxia‐inducible factor‐1*α* (HIF‐1*α*) 50, and (4) peroxisome proliferator‐activated receptor‐*γ* (PPAR‐*γ*) 51 (Fig. 4A).

*XOR* +/− mice showed defective production and secretion of milk fat droplets that impair the lactation process 52. Indeed, XOR has been demonstrated to be essential for the regulation of milk lipid formation and secretion in the mouse mammary gland 53. *XOR* −/− mice also had a defective induction of COX‐2, associated with renal dysplasia, probably because of the uric acid lack 47. Moreover, these mice had an accumulation of triglycerides with injury of renal tubules and interstitial fibrosis possibly due to increased epithelial–mesenchymal transition 54. The amount of adipose mass was proportional to XOR expression by comparing *XOR* −/−, wild‐type, and obese *ob*/*ob* mice, accrediting XOR as a regulator of adipogenesis 51. Additionally, estrogen‐dependent XOR expression and the conversion of XDH to XO were associated with the orthokeratinization pathway of differentiation in the vaginal epithelium of ovariectomized mice 55.

In mammary epithelial cell lines with weak XOR expression and high migratory capacity, the overexpression of XOR cDNA reduced COX‐2 expression and inhibited cell migration in vitro. Conversely, in cell lines with high XOR expression and weak migratory capacity, XOR inhibition promoted increased levels of COX‐2 protein and cell migration in vitro, suggesting a potential role for XOR in suppressing the invasiveness of breast cancer through the regulation of COX‐2 41. XOR expression was downregulated by estrogens, which may promote oncogenesis, whereas the differentiation of mammary epithelial cells was associated with XOR activity increase both in vitro and in vivo. Low XOR expression contributed to breast cancer aggressiveness and predicted a more rapid time to tumor relapse. Similarly, the inhibition of XOR increased tumor growth in a mouse xenograft model of human breast cancer 56. Thus, XOR may be considered a marker of mammary gland development and differentiation (Fig. 4B).

### Oncogenesis

The reaction products of XOR activity, uric acid and ROS/RNS have been implicated both in the process of oncogenesis, as well as in its prevention (reviewed in 57).

The presence of XOR occurs in bladder tissue, and the oxidase form of XOR could catalyze the metabolic activation of a bladder carcinogen, supporting the hypothesis that such activation could occur in vivo 58. Accordingly, the colon tumorigenesis, induced in rats by azo‐dye feeding, was prevented by the oral administration of a XOR inhibitor 59.

Alcohol intake is associated with an increased risk of breast cancer, and a pathogenetic role has been suggested for XOR because acetaldehyde and NADH, both produced by the metabolism of alcohol, can be substrates for XOR, resulting in ROS formation. Moreover, the age‐induced diminished antioxidant defenses and iron accumulation in breast tissue may increase ROS production with the risk of DNA damage and carcinogenic mutations (reviewed in 60). Such in situ bioactivation was shown in breast tissue from postlactation rats, demonstrating the ability of XOR to catalyze ethanol to acetaldehyde and free radicals, which may exert a transforming action 61. The role of XOR‐derived ROS in oncogenesis has been suggested by in vitro study, showing that *hXOR* is targeted by tumor suppressors which repress *hXOR* expression by binding to its promoter 62. The XOR mRNA levels are normally low in most of the tissues because of the repressing regulation at the transcriptional level 63.

A low activity of various oxidative enzymes, including XOR, was correlated with cell proliferation, and the proposed hypothesis was that a low level of free oxygen radicals may promote cancer cell growth 10. However, ROS‐mediated NF*κ*B activation was suggested to increase the expression of genes involved in angiogenesis and metastasis in human cancer 64. XOR‐produced ROS upregulated HIF‐1*α* expression 49. HIF‐1*α* together with activated NF*κ*B, contributes to cancer‐associated inflammatory signaling, as well as to tumor progression by activating angiogenesis, migration, and invasion (reviewed in 65).

In intestinal epithelial cells, phlogistic NF‐*κ*B signaling can induce dedifferentiation into tumor‐initiating cells. The chemical and biological mechanisms involved in the etiology of inflammation‐induced colon cancer were analyzed in correlation with the availability of ROS and RNS. The hypothesis was formulated that these oxidants, produced during inflammation by various sources, including phagocyte XOR, induce oxidative damage to DNA, generating base lesions, strand breaks, genomic aberrations, and chromosomal rearrangements. As a consequence, XOR products can contribute to genetic and epigenetic changes leading to mutation, tumor initiation, and promotion that are responsible for neoplastic transformation through activation of oncogenes and/or inactivation of tumor suppressors (reviewed in 66).

### Controversial role of uric acid

High serum uric acid levels were associated with a short survival time in 118 terminally ill patients with cancer in various organs, including lung and abdominal viscera as primary sites and liver, lung, bone, and brain as metastatic sites 67. Additionally, the serum uric acid level and XOR activity were markedly increased in patients during hepatocarcinoma progression from the lower to higher stages 68. Accordingly, the inhibition of XOR activity improved the outcome in a rat model of cancer cachexia, suggesting a role for XOR‐produced ROS in inflammatory signaling and tissue wasting 69. Thus, both an increment in cell damage and a deterioration of renal function could be responsible for hyperuricemia in end‐stage cancer patients.

Uric acid lowered E‐cadherin levels in cultured NRK renal tubular cells either by decreasing its synthesis and increasing its degradation. Experimental hyperuricemia in rats was associated with the downregulation of E‐cadherin expression and increase in alpha‐smooth muscle actin expression, indicating an epithelial–mesenchymal transition. These phenotypic alterations, which occur during tumor progression toward malignity, were inhibited by allopurinol 70.

The plasma level of uric acid was significantly higher in patients with squamous cell carcinoma of the head and neck region than in control subjects. Uricemia was also observed to increase with staging, thus behaving as a diagnostic and prognostic marker of head and neck cancers 71. Hyperuricemia may contribute to cancer pathogenesis by promoting an increased intracellular concentration of uric acid, with consequent pro‐inflammatory effects due to ROS and RNS generation and COX‐2 activation. After the neoplastic transformation, the scavenger action of extracellular uric acid may protect cancer cells from oxidative stress, thus contributing to their survival (reviewed in 72). In addition, the uric acid can reduce the tumor surveillance by the immune system as it is able to stimulate the chemotaxis of mesenchymal stromal human cells, which are associated with tumor progression and metastasis and can promote the differentiation of immunosuppressive IDO+/IL10+ lymphocytes 73. Accordingly, an elevated uric acid level in plasma at the time of diagnosis was a negative prognostic marker in a cohort of 466 pancreatic cancer patients 74. Also, in two cohort studies including 83,683 men and 28,613 women with a median follow‐up of 13.6 and 15.2 years, respectively, high serum uric acid concentrations were associated with increased risk of cancer mortality 75, 76. Moreover, the above wide cohort of male patients was further analyzed, investigating the association of serum uric acid and cancer incidence, by using a nonlinear statistical test (penalized splines in extended Cox‐type additive hazard regression). The results of the study evidenced the “J‐shaped” nature of the relationship between serum uric acid and cancer incidence and indicated a positive dose–response association between these parameters for serum uric acid above the normal level 77.

In contrast, the antioxidant activity of uric acid could exert a contrasting action against free‐radical oncogenesis. The incidence of lung cancer (1015 cases) in cigarette smokers was inversely related to the serum uric acid level 78. The hypothesis has been formulated that the loss of uricase during primate evolution may have had a crucial role in lengthening life‐span because of the scavenger activity of uric acid (reviewed in 79) with the consequent reduction in age‐specific cancer rates 80. A link has been demonstrated between the tumor suppression by p53 and antioxidant function of uric acid through its transporter SLC2A9, whose expression is induced by oxidative stress and is dependent on p53. An increased ROS level was reported by inhibiting SLC2A9 with small‐interfering RNA or because of p53 loss. Thus, the poorer prognosis associated with decreased SLC2A9 expression that was described in several cancer types suggests that the p53‐SLC2A9 system could prevent the ROS‐associated damage contributing to cancer development 81.

Elevated levels of uric acid have been associated with better survival in various malignancies. In colorectal cancer patients, a better prognosis was associated with an elevated level of serum uric acid, suggesting this as an efficient predictor of survival 82. The prognostic relevance of preoperative uric acid plasma levels to cancer‐specific survival was evaluated in 357 soft‐tissue sarcoma patients who underwent tumor resection. Conclusions similar to those above reported were suggested by the positive clinical outcome, which was associated with elevated uric acid serum levels 83. The protection against cancer mortality given by elevated serum uric acid levels was demonstrated by a large general population‐based cohort study with 4350 patients who died because of cancer, in particular, cancer localized to the lung, colorectal intestine, and prostate 84.

## Conclusion

In most tissues, *hXOR* is normally downregulated at the transcriptional level, except in the breast, liver, gastrointestinal tract, and kidney. Differentiation is associated with the expression of a high level of XOR protein in these organs, whereas the lack of differentiation, which occurs in malignant neoplasms, corresponds to a low concentration of XOR. In addition, in the case of the mammary gland, XOR plays a structural role and may be considered a marker of development and differentiation. By activating PPAR‐*γ*, XOR promotes cell differentiation and produces an antitumorigenic and antiproliferative action. Moreover, the low XOR activity, together with an increase in purine biosynthesis, provides a selective advantage to the growth of tumor tissue. Indeed, XOR expression was negatively associated with a high malignity grade and a worse prognosis in cancer patients.

The elevated XOR activity level observed in the plasma of patients with different types of cancer may be related to the inflammatory response elicited by the tissue damage induced by tumor growth. A similar reaction is possibly responsible for an elevation of XOR expression in usually low‐expressing tissues; if this interpretation is correct, the level of XOR expression should be positively associated with a worse outcome in these cancer patients.

The role of XOR activity and its products in oncogenesis has been suggested. XOR in its oxidase form could directly catalyze the metabolic activation of carcinogenic substances. In most cases, XOR has an indirect tumorigenic action by producing ROS and RNS, which may activate proflogistic, antiapoptotic, and angiogenic target genes, by NF‐kB and HIF‐1*α* upregulation. Uric acid promotes inflammation, cell proliferation, and migration by inducing COX‐2 expression. However, the role of uric acid in cancer is still debated. The antioxidant function of circulating uric acid justifies the overall protective role against neoplasia, although the elevation of its plasma level is typical of the end stages of oncologic illness that is associated with tumor lysis syndrome.

Current knowledge does not allow to suggest the use of available XOR inhibitors to prevent both ROS/RNS formation and oncogenesis. However, XOR inhibitors have a wide pharmacological use in hyperuricemic patients with metabolic syndrome, renal diseases or tumor lysis syndrome. Thus, a large amount of data will be available in the future for further studies to understand the correlation between XOR activity, reflected by the serum uric acid level, and cancer incidence and prognosis. The results of such meta‐analysis will possibly discriminate the cases in which the control of the uricemia will be helpful to improve the outcomes of tumor illness. In addition, a conditional knockout strategy in mice would be useful to test the differential role of XOR and uric acid in preclinical model of tumorigenesis and cancer progression.

## Conflict of Interest

None declared.
